# Supplementary material for: Pyruvate Dehydrogenase Kinase 1 inhibition mediated oxidative phosphorylation enhancement in cartilage promotes osteoarthritis progression
Source: BMC Musculoskelet Disord. 2023 Jul 20;24:597. doi: 10.1186/s12891-023-06585-6 (PMC10357736; doi:10.1186/s12891-023-06585-6)
Supplement: Supplementary file 1 — Additional file 1. [file 12891_2023_6585_MOESM1_ESM.zip › KEGG.Gsea.1653622432464/KEGG_DILATED_CARDIOMYOPATHY.html]

Details for gene set KEGG\_DILATED\_CARDIOMYOPATHY[GSEA]

|  || Dataset | 55457\_collapsed\_to\_symbols.cls.cls #OA\_versus\_normal.cls.cls #OA\_versus\_normal\_repos |
| Phenotype | cls.cls#OA\_versus\_normal\_repos |
| Upregulated in class | OA |
| GeneSet | KEGG\_DILATED\_CARDIOMYOPATHY |
| Enrichment Score (ES) | 0.1516647 |
| Normalized Enrichment Score (NES) | 0.67533964 |
| Nominal p-value | 0.9917808 |
| FDR q-value | 1.0 |
| FWER p-Value | 1.0 |
Table: GSEA Results Summary

  

Fig 1: Enrichment plot: KEGG\_DILATED\_CARDIOMYOPATHY      
 Profile of the Running ES Score & Positions of GeneSet Members on the Rank Ordered List

  

| SYMBOL | TITLE | RANK IN GENE LIST | RANK METRIC SCORE | RUNNING ES | CORE ENRICHMENT || 1 | SGCA | sarcoglycan alpha [Source:HGNC Symbol;Acc:HGNC:10805] | 196 | 0.824 | 0.0184 | Yes |
| 2 | SGCB | sarcoglycan beta [Source:HGNC Symbol;Acc:HGNC:10806] | 687 | 0.597 | 0.0046 | Yes |
| 3 | PRKACB | protein kinase cAMP-activated catalytic subunit beta [Source:HGNC Symbol;Acc:HGNC:9381] | 771 | 0.576 | 0.0217 | Yes |
| 4 | RYR2 | ryanodine receptor 2 [Source:HGNC Symbol;Acc:HGNC:10484] | 828 | 0.561 | 0.0403 | Yes |
| 5 | CACNA2D3 | calcium voltage-gated channel auxiliary subunit alpha2delta 3 [Source:HGNC Symbol;Acc:HGNC:15460] | 1228 | 0.471 | 0.0284 | Yes |
| 6 | SGCG | sarcoglycan gamma [Source:HGNC Symbol;Acc:HGNC:10809] | 1284 | 0.461 | 0.0430 | Yes |
| 7 | ADCY7 | adenylate cyclase 7 [Source:HGNC Symbol;Acc:HGNC:238] | 1408 | 0.442 | 0.0514 | Yes |
| 8 | CACNA2D2 | calcium voltage-gated channel auxiliary subunit alpha2delta 2 [Source:HGNC Symbol;Acc:HGNC:1400] | 1431 | 0.439 | 0.0677 | Yes |
| 9 | ADCY1 | adenylate cyclase 1 [Source:HGNC Symbol;Acc:HGNC:232] | 1589 | 0.415 | 0.0724 | Yes |
| 10 | SGCD | sarcoglycan delta [Source:HGNC Symbol;Acc:HGNC:10807] | 1781 | 0.387 | 0.0733 | Yes |
| 11 | ITGA4 | integrin subunit alpha 4 [Source:HGNC Symbol;Acc:HGNC:6140] | 1854 | 0.379 | 0.0832 | Yes |
| 12 | TNF | tumor necrosis factor [Source:HGNC Symbol;Acc:HGNC:11892] | 2098 | 0.350 | 0.0786 | Yes |
| 13 | IGF1 | insulin like growth factor 1 [Source:HGNC Symbol;Acc:HGNC:5464] | 2286 | 0.328 | 0.0774 | Yes |
| 14 | ACTC1 | actin alpha cardiac muscle 1 [Source:HGNC Symbol;Acc:HGNC:143] | 2331 | 0.323 | 0.0872 | Yes |
| 15 | MYL2 | myosin light chain 2 [Source:HGNC Symbol;Acc:HGNC:7583] | 2376 | 0.317 | 0.0967 | Yes |
| 16 | TNNC1 | "troponin C1, slow skeletal and cardiac type [Source:HGNC Symbol;Acc:HGNC:11943]" | 2396 | 0.315 | 0.1081 | Yes |
| 17 | ITGA1 | integrin subunit alpha 1 [Source:HGNC Symbol;Acc:HGNC:6134] | 2421 | 0.311 | 0.1190 | Yes |
| 18 | TGFB1 | transforming growth factor beta 1 [Source:HGNC Symbol;Acc:HGNC:11766] | 2490 | 0.303 | 0.1261 | Yes |
| 19 | TTN | titin [Source:HGNC Symbol;Acc:HGNC:12403] | 2492 | 0.303 | 0.1384 | Yes |
| 20 | MYH6 | myosin heavy chain 6 [Source:HGNC Symbol;Acc:HGNC:7576] | 2555 | 0.297 | 0.1457 | Yes |
| 21 | CACNA1F | calcium voltage-gated channel subunit alpha1 F [Source:HGNC Symbol;Acc:HGNC:1393] | 2726 | 0.278 | 0.1438 | Yes |
| 22 | ITGB5 | integrin subunit beta 5 [Source:HGNC Symbol;Acc:HGNC:6160] | 2823 | 0.267 | 0.1473 | Yes |
| 23 | CACNG4 | calcium voltage-gated channel auxiliary subunit gamma 4 [Source:HGNC Symbol;Acc:HGNC:1408] | 3065 | 0.244 | 0.1384 | Yes |
| 24 | MYH7 | myosin heavy chain 7 [Source:HGNC Symbol;Acc:HGNC:7577] | 3161 | 0.234 | 0.1406 | Yes |
| 25 | DAG1 | dystroglycan 1 [Source:HGNC Symbol;Acc:HGNC:2666] | 3203 | 0.230 | 0.1468 | Yes |
| 26 | EMD | emerin [Source:HGNC Symbol;Acc:HGNC:3331] | 3404 | 0.211 | 0.1398 | Yes |
| 27 | TPM3 | tropomyosin 3 [Source:HGNC Symbol;Acc:HGNC:12012] | 3484 | 0.204 | 0.1420 | Yes |
| 28 | MYL3 | myosin light chain 3 [Source:HGNC Symbol;Acc:HGNC:7584] | 3529 | 0.199 | 0.1467 | Yes |
| 29 | DES | desmin [Source:HGNC Symbol;Acc:HGNC:2770] | 3570 | 0.197 | 0.1517 | Yes |
| 30 | PLN | phospholamban [Source:HGNC Symbol;Acc:HGNC:9080] | 3710 | 0.184 | 0.1484 | No |
| 31 | CACNG2 | calcium voltage-gated channel auxiliary subunit gamma 2 [Source:HGNC Symbol;Acc:HGNC:1406] | 3819 | 0.174 | 0.1471 | No |
| 32 | MYBPC3 | myosin binding protein C3 [Source:HGNC Symbol;Acc:HGNC:7551] | 3926 | 0.162 | 0.1454 | No |
| 33 | CACNA1S | calcium voltage-gated channel subunit alpha1 S [Source:HGNC Symbol;Acc:HGNC:1397] | 4031 | 0.153 | 0.1436 | No |
| 34 | TPM1 | tropomyosin 1 [Source:HGNC Symbol;Acc:HGNC:12010] | 4220 | 0.135 | 0.1344 | No |
| 35 | CACNA2D1 | calcium voltage-gated channel auxiliary subunit alpha2delta 1 [Source:HGNC Symbol;Acc:HGNC:1399] | 4747 | 0.091 | 0.0971 | No |
| 36 | CACNG1 | calcium voltage-gated channel auxiliary subunit gamma 1 [Source:HGNC Symbol;Acc:HGNC:1405] | 4773 | 0.089 | 0.0988 | No |
| 37 | ADCY9 | adenylate cyclase 9 [Source:HGNC Symbol;Acc:HGNC:240] | 5191 | 0.057 | 0.0686 | No |
| 38 | TNNI3 | "troponin I3, cardiac type [Source:HGNC Symbol;Acc:HGNC:11947]" | 5549 | 0.029 | 0.0419 | No |
| 39 | DMD | dystrophin [Source:HGNC Symbol;Acc:HGNC:2928] | 5605 | 0.023 | 0.0386 | No |
| 40 | TGFB3 | transforming growth factor beta 3 [Source:HGNC Symbol;Acc:HGNC:11769] | 6293 | -0.027 | -0.0139 | No |
| 41 | CACNA1C | calcium voltage-gated channel subunit alpha1 C [Source:HGNC Symbol;Acc:HGNC:1390] | 6367 | -0.033 | -0.0182 | No |
| 42 | ADCY8 | adenylate cyclase 8 [Source:HGNC Symbol;Acc:HGNC:239] | 6646 | -0.053 | -0.0378 | No |
| 43 | CACNB3 | calcium voltage-gated channel auxiliary subunit beta 3 [Source:HGNC Symbol;Acc:HGNC:1403] | 6753 | -0.059 | -0.0436 | No |
| 44 | TGFB2 | transforming growth factor beta 2 [Source:HGNC Symbol;Acc:HGNC:11768] | 6985 | -0.079 | -0.0584 | No |
| 45 | ITGAV | integrin subunit alpha V [Source:HGNC Symbol;Acc:HGNC:6150] | 7067 | -0.085 | -0.0612 | No |
| 46 | LAMA2 | laminin subunit alpha 2 [Source:HGNC Symbol;Acc:HGNC:6482] | 7392 | -0.110 | -0.0820 | No |
| 47 | TNNT2 | "troponin T2, cardiac type [Source:HGNC Symbol;Acc:HGNC:11949]" | 7441 | -0.114 | -0.0811 | No |
| 48 | CACNB1 | calcium voltage-gated channel auxiliary subunit beta 1 [Source:HGNC Symbol;Acc:HGNC:1401] | 7589 | -0.126 | -0.0874 | No |
| 49 | ITGA9 | integrin subunit alpha 9 [Source:HGNC Symbol;Acc:HGNC:6145] | 7785 | -0.140 | -0.0969 | No |
| 50 | ITGA2 | integrin subunit alpha 2 [Source:HGNC Symbol;Acc:HGNC:6137] | 7857 | -0.145 | -0.0965 | No |
| 51 | ITGB7 | integrin subunit beta 7 [Source:HGNC Symbol;Acc:HGNC:6162] | 7873 | -0.146 | -0.0916 | No |
| 52 | TPM4 | tropomyosin 4 [Source:HGNC Symbol;Acc:HGNC:12013] | 7974 | -0.153 | -0.0932 | No |
| 53 | TPM2 | tropomyosin 2 [Source:HGNC Symbol;Acc:HGNC:12011] | 8156 | -0.167 | -0.1005 | No |
| 54 | ADRB1 | adrenoceptor beta 1 [Source:HGNC Symbol;Acc:HGNC:285] | 8212 | -0.172 | -0.0977 | No |
| 55 | ADCY3 | adenylate cyclase 3 [Source:HGNC Symbol;Acc:HGNC:234] | 8572 | -0.199 | -0.1176 | No |
| 56 | CACNG3 | calcium voltage-gated channel auxiliary subunit gamma 3 [Source:HGNC Symbol;Acc:HGNC:1407] | 8677 | -0.207 | -0.1172 | No |
| 57 | GNAS | GNAS complex locus [Source:HGNC Symbol;Acc:HGNC:4392] | 8929 | -0.226 | -0.1275 | No |
| 58 | ATP2A2 | ATPase sarcoplasmic/endoplasmic reticulum Ca2+ transporting 2 [Source:HGNC Symbol;Acc:HGNC:812] | 9015 | -0.233 | -0.1246 | No |
| 59 | ITGA6 | integrin subunit alpha 6 [Source:HGNC Symbol;Acc:HGNC:6142] | 9112 | -0.240 | -0.1223 | No |
| 60 | ITGA10 | integrin subunit alpha 10 [Source:HGNC Symbol;Acc:HGNC:6135] | 9296 | -0.256 | -0.1261 | No |
| 61 | ITGB8 | integrin subunit beta 8 [Source:HGNC Symbol;Acc:HGNC:6163] | 9442 | -0.268 | -0.1265 | No |
| 62 | ITGB6 | integrin subunit beta 6 [Source:HGNC Symbol;Acc:HGNC:6161] | 9614 | -0.284 | -0.1282 | No |
| 63 | ADCY2 | adenylate cyclase 2 [Source:HGNC Symbol;Acc:HGNC:233] | 9715 | -0.293 | -0.1240 | No |
| 64 | ITGA7 | integrin subunit alpha 7 [Source:HGNC Symbol;Acc:HGNC:6143] | 9724 | -0.294 | -0.1126 | No |
| 65 | CACNA1D | calcium voltage-gated channel subunit alpha1 D [Source:HGNC Symbol;Acc:HGNC:1391] | 9867 | -0.308 | -0.1110 | No |
| 66 | CACNB2 | calcium voltage-gated channel auxiliary subunit beta 2 [Source:HGNC Symbol;Acc:HGNC:1402] | 9967 | -0.318 | -0.1058 | No |
| 67 | CACNB4 | calcium voltage-gated channel auxiliary subunit beta 4 [Source:HGNC Symbol;Acc:HGNC:1404] | 9992 | -0.320 | -0.0945 | No |
| 68 | ITGB4 | integrin subunit beta 4 [Source:HGNC Symbol;Acc:HGNC:6158] | 10159 | -0.337 | -0.0937 | No |
| 69 | ITGB3 | integrin subunit beta 3 [Source:HGNC Symbol;Acc:HGNC:6156] | 10310 | -0.352 | -0.0910 | No |
| 70 | PRKACG | protein kinase cAMP-activated catalytic subunit gamma [Source:HGNC Symbol;Acc:HGNC:9382] | 10466 | -0.370 | -0.0880 | No |
| 71 | PRKACA | protein kinase cAMP-activated catalytic subunit alpha [Source:HGNC Symbol;Acc:HGNC:9380] | 10611 | -0.384 | -0.0835 | No |
| 72 | LMNA | lamin A/C [Source:HGNC Symbol;Acc:HGNC:6636] | 10946 | -0.424 | -0.0922 | No |
| 73 | ITGA8 | integrin subunit alpha 8 [Source:HGNC Symbol;Acc:HGNC:6144] | 11046 | -0.437 | -0.0820 | No |
| 74 | PRKX | protein kinase X-linked [Source:HGNC Symbol;Acc:HGNC:9441] | 11634 | -0.522 | -0.1065 | No |
| 75 | ITGA2B | integrin subunit alpha 2b [Source:HGNC Symbol;Acc:HGNC:6138] | 11645 | -0.524 | -0.0858 | No |
| 76 | ADCY6 | adenylate cyclase 6 [Source:HGNC Symbol;Acc:HGNC:237] | 12005 | -0.594 | -0.0895 | No |
| 77 | SLC8A1 | solute carrier family 8 member A1 [Source:HGNC Symbol;Acc:HGNC:11068] | 12192 | -0.642 | -0.0778 | No |
| 78 | ITGA5 | integrin subunit alpha 5 [Source:HGNC Symbol;Acc:HGNC:6141] | 12474 | -0.737 | -0.0695 | No |
| 79 | ITGB1 | integrin subunit beta 1 [Source:HGNC Symbol;Acc:HGNC:6153] | 12566 | -0.780 | -0.0447 | No |
| 80 | ACTG1 | actin gamma 1 [Source:HGNC Symbol;Acc:HGNC:144] | 12613 | -0.806 | -0.0153 | No |
| 81 | ACTB | actin beta [Source:HGNC Symbol;Acc:HGNC:132] | 12757 | -0.920 | 0.0112 | No |
Table: GSEA details [plain text format]

  

Fig 2: KEGG\_DILATED\_CARDIOMYOPATHY      
 Blue-Pink O' Gram in the Space of the Analyzed GeneSet

  

Fig 3: KEGG\_DILATED\_CARDIOMYOPATHY: Random ES distribution      
 Gene set null distribution of ES for **KEGG\_DILATED\_CARDIOMYOPATHY**
